# Synthesis and Characterization of a Biopolymer Pectin/Ethanolic Extract from Olive Mill Wastewater: In Vitro Safety and Efficacy Tests on Skin Wound Healing

**DOI:** 10.3390/ijms242015075

**Published:** 2023-10-11

**Authors:** Francesca Aiello, Rocco Malivindi, Marisa Francesca Motta, Pasquale Crupi, Rosa Nicoletti, Cinzia Benincasa, Maria Lisa Clodoveo, Vittoria Rago, Umile Gianfranco Spizzirri, Donatella Restuccia

**Affiliations:** 1 Department of Pharmacy, Health and Nutritional Sciences, University of Calabria, 87036 Rende, Italy; rocco.malivindi@unical.it (R.M.); mottamarisaf@icloud.com (M.F.M.); vittoria.rago@unical.it (V.R.); g.spizzirri@unical.it (U.G.S.); donatella.restuccia@unical.it (D.R.); 2 Interdisciplinary Department of Medicine, University Aldo Moro Bari, 70121 Bari, Italy; pasquale.crupi@uniba.it (P.C.); marialisa.clodoveo@uniba.it (M.L.C.); 3 Council for Agricultural Research and Economics (CREA), Research Centre for Olive, Fruit and Citrus Crops, Via Settimio Severo 83, 87036 Rende, Italy; rosa.nicoletti@crea.gov.it (R.N.); cinzia.benincasa@crea.gov.it (C.B.); 4 DICEM—Department of European and Mediterranean Cultures: Architecture, Environment, and Cultural Heritage, University of Basilicata, Matera, Via Lanera, 20, 75100 Matera, Italy; 5 Ionian Department of Law, Economics and Environment, University of Bari Aldo Moro, 74123 Taranto, Italy

**Keywords:** olive mill wastewater, ultra-sound-assisted extraction, antioxidant molecules, wound healing

## Abstract

Wound-healing delay is one of the major problems of type 2 diabetes, representing also a clinical emergency in non-healing chronic wounds. Natural antioxidants show interesting wound-healing properties, including those extracted from waste derived from olive oil production. Olive mill wastewater is one of the main by-products of the olive oil-making process, and it is rich in high-value secondary metabolites, mainly hydroxytyrosol. We proposed an eco-friendly extraction method, employing both ultrasound-assisted and Soxhlet techniques and ethanol as a solvent, to recover valuable molecules from Roggianella cv (*Olea europea* L.) olive mill wastewater, which was further entrapped in a pectin polymer via an enzymatic reaction using porcine pancreatic lipase. Pectin, in combination with other substances, promoted and accelerated wound healing and demonstrated good potential to produce a biomedical conjugate for wound treatment. The antioxidant activity of the extracts and conjugate were evaluated against lipophilic (IC_50_ equal to 0.152 mg mL^−1^) and hydrophilic (IC_50_ equal to 0.0371 mg mL^−1^) radical species as well as the in vitro cytotoxicity via NRU, h-CLAT, and a wound-healing scratch assay and assessment. The pectin conjugate did not exert hemolytic effects on the peripheral blood, demonstrating interesting wound-healing properties due to its ability to stimulate cell proliferation in a dose-dependent manner.

## 1. Introduction

The skin integrity is essential to avoid external stress. Injuries, illness, and burns can damage its intactness, causing wounds [1]. The healing process involves overlapping steps: inflammation, proliferation, and remodeling, leading finally to wound closure (acute wounds). On the contrary, chronic wounds are defined as non-healing wounds due to the impossibility of restoring the skin barrier, which is caused by an excessive neutrophil infiltration generating a permanent high-inflammatory tissue; at the same time, matrix metalloproteinases are found to be abundant as well, leading to long-time healing. These chronic wounds still represent a clinical emergency [2]. The current treatment methodologies are wound dressing, vacuum-assisted closure procedures, photo biomodulation, hyperbaric oxygen, immune-modulatory biomaterials, the application of bacteriophages, angiogenesis promotion, and antioxidative and antibacterial nanomaterials [3]. However, all these protocols are expensive, limited in their efficacy, and time consuming. For this reason, the discovery of new therapeutic alternatives seems to be necessary. Natural products, especially antioxidants [4,5] and their derivatives [6,7,8,9], can offer similar, if not better, biological activity than many synthetic drugs that are used in healing chronic wounds, thanks to their ability to reduce the inflammation in the wound region as well as the bacterial proliferation. Furthermore, considering the oxidative stress that is stated in chronic wounds, an antioxidant agent could be helpful during the wound-healing evolution. In this context, food production wastes could be also a valuable source of secondary metabolites supporting the wound-healing process [10]. 

Considering that drug carriers are mostly used in wound-healing applications [11,12], in this experimental work, olive mill wastewater (OMW) was proposed as a source of active molecules that are opportunely linked to a biopolymer. With about 3 million tons produced last year and a global market valued at USD 12,989,630,000, olive oil is a pillar of Mediterranean countries, mostly Spain, Italy, and Greece [13]. Nevertheless, during production, olive oil generates huge amounts of different wastes (i.e., pruning residues, stones, olive mill wastewaters, and pomace). Among them, OMW and olive pomace represent about 35–45% of the processed drupes, representing a severe environmental burden in terms of both quantities (30 million m^3^ per year and 2 million tons per year, respectively) and polluting capacity (pH, chemical and biological oxygen demand, etc.) [14]. The polyphenols that are present in OMW have been already investigated for the treatment of skin disorders. Recently, the lipophenol hydroxytyrosil oleate (HtyOle) was recovered from olive pomace and OMW, to be evaluated for their antioxidant capacity in human keratinocytes. The formation of reactive oxygen species (ROS) and malondialdehyde (MDA), as well as the activity of glutathione-S-transferase (GST) and superoxide dismutase (SOD), were reduced by HtyOle [15].

In OMW, the main compounds are derived from hydroxylation using benzoic acid (C_6_–C_1_) or cinnamic acid (C_6_–C_3_) (phenylpropanoid family). Hydroxytyrosol (HT) and tyrosol are frequently found in OMW together with caffeic and ferulic acids and more complex phenolic compounds, such as verbascoside, oleuropein, and isomers/derivatives. Furthermore, among the phenolic compounds detected in dried mill wastewater by liquid chromatography–tandem mass spectrometry (HPLC-MS/MS), the most abundant were apigenin (9.55 mg/kg dry weight), *p*-coumaric acid (5.01 mg/kg dry weight), diosmetin (3.58 mg/kg dry weight), hydroxytyrosil oleate (564 mg/kg dry weight), luteolin (62.38 mg/kg dry weight), luteolin-7-*O*-glucoside (88.55 mg/kg dry weight), luteolin-4-*O*-glucoside (11.48 mg/kg dry weight), and rutin (48.52 mg/kg dry weight) [16]. The catechol function confers strong antioxidant activity to these compounds, which is exerted either by chelating metals that participate in the generation of free radicals or by direct neutralization of the free radicals by transformation into a stable quinone final product [17].

In this research, a lyophilized OWM has been successfully extracted using Soxhlet or ultrasound-assisted methodologies by employing solvents at different polarity (ethanol, acetone, dichloromethane, *n*-hexane, and hydroalcoholic mixture). Colorimetric tests, performed to evaluate the antioxidant potential of the extracts, allowed to individuate that the ethanolic one is the extract richest in phenolic compounds. Additionally, spectroscopic methodologies (HPLC/MS/MS, and NMR) revealed the presence of several interesting metabolites sharing the 3,4-dihydroxyphenilethyl moiety, which is typical of the HY. Several types of scientific evidence highly recommended HY as a bioactive compound to be used as a pharmaceutical product for wound care applications as HY showed useful proangiogenic, antioxidative, antiaging, anti-inflammatory, and antimicrobial effects [17,18]. For all these reasons, we aim to develop a functional pectin biopolymer as a new tool for wound-healing applications. The OH in the active compounds of ethanolic extract can be transesterified with the COOCH_3_ moieties of the pectin by using porcine pancreatic lipase (PPL) as a green and recyclable catalyst. The functional pectin biopolymer was characterized using the ESI MS/MS and NMR. To reveal the better wound healing properties endowed by the functional polymer, in vitro assays were carried out also to demonstrate the absence of hemolysis activity. 

## 2. Results and Discussion

### 2.1. Antioxidant Evaluation of ELAVF Extracts

OMW is an aqueous suspension rich in organic and inorganic compounds, such as tannins, pectins as well as phenolic molecules ranging from 0.5 to 24 g/OMW and representing about 98% (*w*/*w*) of the phenols that are usually found in the drupe [19]. The vegetal by-product was freeze-dried producing a powder that is easy to handle (ELAVF). ELAVF displayed remarkable biological properties related to a high concentration of available phenolic groups (TPC) (75.0 mg CT/g)—mainly flavonoid molecules (34.0 mg CT/g) and phenolic acids (50.8 mg CT /g) [20]. 

These compounds can impart OMW suitable antioxidant properties, which have been deeply investigated by scavenger activity measures against hydrophilic and lipophilic radical species. Specifically, the inhibition kinetic studies returned an IC_50_ value against the ABTS radical (0.019 mg mL^−1^) that was nearly five times lower compared to the activity against lipophilic species (DPPH). 

The experimental parameters applied during the vegetable matrix extraction (i.e., pH, time, type of solvent, and concentration) are critical parameters for the activity of the phenols-containing extracts. Phenolic compounds are usually soluble in polar protic solvents such as ethanol or methanol. However, phenolic acids such as gallic, cinnamic, and coumaric acids are soluble in water as well as in dichloromethane or acetone. For these reasons, several studies proposed mixtures consisting of ethanol and water in different proportions for the extraction of bioactive compounds from OMW [21]. ELAVF was subjected to an extraction process by maceration using different solvents with different polarity, including ethanol, acetone, dichloromethane, *n*-hexane and hydroalcoholic solution (10/90 *v*/*v*). To improve the extraction process, a second reflux method, i.e., Soxhlet apparatus, was proposed. It represents a valid alternative method to increase the extraction yields [22]. The extraction conditions used, together with the process yields, expressed as grams of dry substance obtained, and the evaluation of the phenolic profile, are described in Table 1. Particularly, ELAVF5S (52.10%) and ELAVF1S (25.60%) provided higher extraction yields. 

The extraction data analysis showed that the Soxhlet process returned the best results in terms of yields compared to the maceration technique. Colorimetric assays were used to explore the presence of active compounds in the extracts and to correlate them to the antioxidant performances of the matrices. The comparison between the values of the quantities of available phenolic groups, recorded for the different extracts on the basis of a single type of solvent used, appeared higher for the samples obtained using the reflux extraction technique with the exception of the sample obtained using dichloromethane. The obtained extracts were richer in polyphenolic compounds when the extraction process was carried out in the presence of ethanol (94.16 mg CT/g extract, for ELAVF1S), which was followed by acetone (28.98 mg CT/g extract, for ELAVF2S) and the ethanol–water mixture (90/10 *v*/*v*) (22.93 mg CT/g extract, for ELAVF5S). The antioxidant profiles of the extracts were investigated by employing the same colorimetric tests used for the raw matrix. All experiments confirmed TPC results, highlighting as ELAVF1S is the best performed extract. The ethanol extract ELAVF1S showed a greater quantity of available phenolic groups (94.16 mg CT/g of extract) as well as a high amount of phenolic acids and flavonoids, showing percentages equal to 69.9% and 66.0% of the total polyphenols, respectively (Table 2). 

However, the results of the assays relating to the total antioxidant activity of the starting LAVF and ELAVF1S samples are almost equal. Specifically, ELAVF1S showed total antioxidant activity at 1.0 mg CT/g of extract (Table 2) as confirmed by the analysis of the inhibition profiles toward the lipophilic radical DPPH and hydrophilic specie ABTS. The ability of ELAVF1S to inhibit DPPH and ABTS radicals, expressed in terms of IC_50_ (mg mL^−1^), is shown in Table 2. The IC_50_ value of the ELAVF1S sample showed a difference of almost an order of magnitude recorded in the scavenging activity in an aqueous environment compared to the organic one.

### 2.2. HPLC-MS/MS Analysis of ELAVFS1

The chromatographic elution order, deprotonated [M–H]^−^ ions, MS/MS fragmentation patterns, and acquisition parameters for MRM analysis of the identified polyphenols in the ELAVFS1 extracts are listed in Table 3. 

In agreement with our previous report on olive mill wastewater [20], the same compounds including five simple phenols, two hydroxycinnamic acids, and five secoiridoid derivatives were recognized in ELAVF (Table 3). The extraction of polyphenols depends on their diffusion into the extraction solvent, which is determined either by their structure or by their interactions with other matrix components. In this work, ethanol was chosen as the solvent because, relating to lyophilized olive mill wastewater, lower molecular weight polyphenols can be sufficiently extracted with simple alcohols or a hydroalcoholic mixture [23]. 

Overall, the collected data displayed that the HT and its glucoside isomers (7.1 ± 0.6, 21.2 ± 1.9, and 12.1 ± 1.1 μg mL^−1^) were recognized as having the same [M–H]^−^ at *m*/*z* 315. The MS/MS spectra showed two main fragment ions at *m*/*z* 153 and 123, belonging to the compounds recovered at higher concentrations in ethanol. Quinic acid (4.1 ± 0.4 μg mL^−1^), caffeic acid (6.4 ± 0.6 μg mL^−1^), and *p*-coumaric acid (5.9 ± 0.3 μg mL^−1^), identified through their characteristic parent and daughter ions, were also quantified. Finally, a few secoiridoid derivatives, such as verbascoside residue, decarboxymethyl-elenolic acid and its derivatives, and especially oleuropein aglycone derivative (5.9 ± 0.3 μg mL^−1^), were detected (Table 3).

### 2.3. NMR of ELAVF

**Figure 1:**
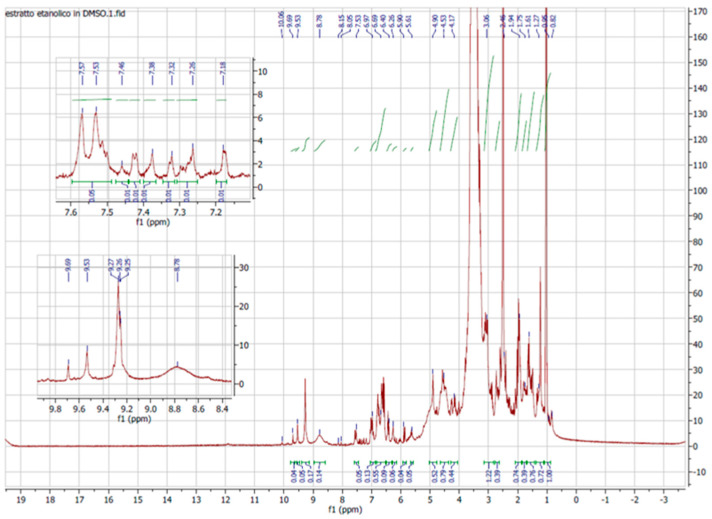
^1^H NMR spectra of ELAVFS1 in DMSO.

**Figure 2:**
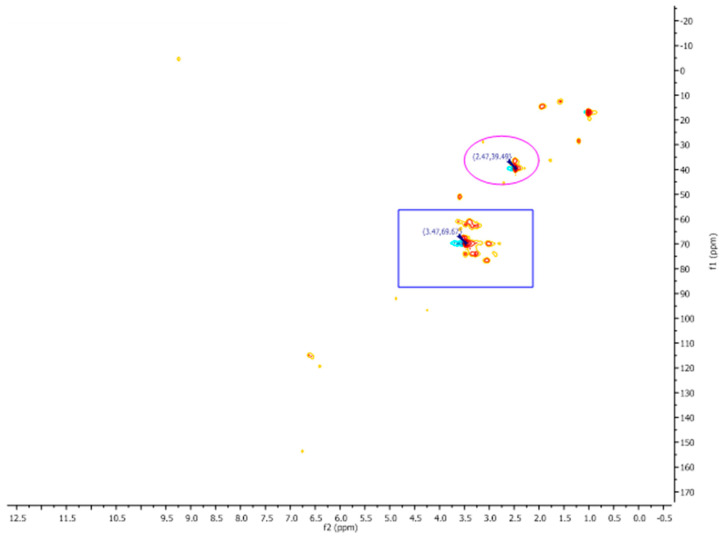
^13^CHSQC spectra of ELAVFS1 in DMSO.

**Figure 3:**
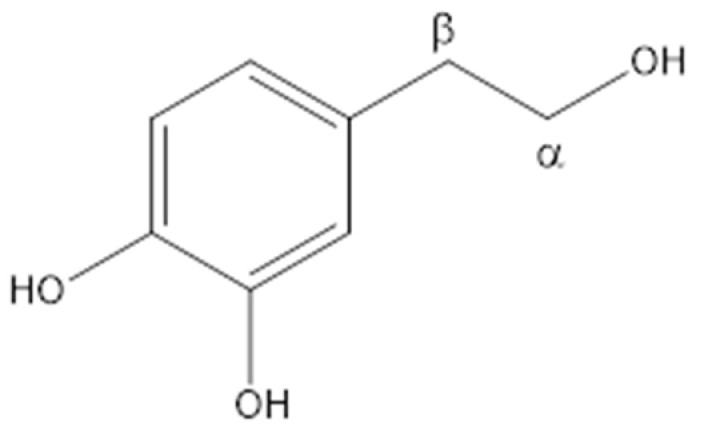
Hydroxytyrosol chemical structure.

The 1D and 2D NMR spectra of ELAVF1S were acquired using DMSO as the solvent due to the total insolubility of the extract in other deuterated solvents. Unfortunately, the key signals of the hydroxythyrosol tail and structural corelated analogs detected by LC-MS/MS fell in the solvent cone (2.5, 3.5 ppm, respectively; see Figure 1), resulting in them being drowned out. The opposite was found for the aromatic signals falling in the spectral windows between 6.0 and 7.0 ppm and the phenolic OH at value higher than 8.00 ppm, which is in accordance with the literature data. However, to better assign the signal related to the catechol ethanolic scaffold, a 2D ^13^CHSQC spectrum was recorded (Figure 2). It revealed a good correspondence between the signals at δ 2.47 ppm ^1^H NMR with 39.49 ppm of ^13^CNMR (βC) and 3.47 ppm with 69.67 ppm (αC), and in a wide range of these ppm, see purple circle and blue rectangle, confirming the presence of compounds endowing the catechol ethanolic (Figure 3), as observed in LC-MS analyses.

### 2.4. NMR and ESI-MS/MS Analysis of PELAVF1S 

**Figure 4:**
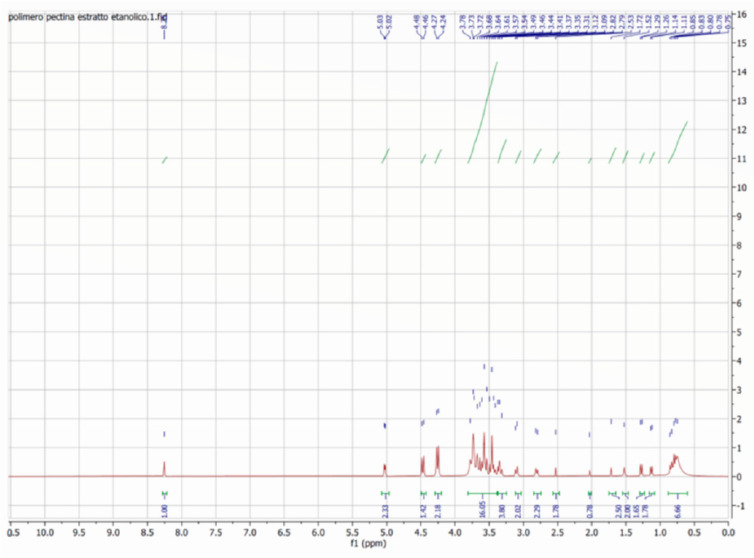
^1^H NMR spectra of PELAVFS1 in D_2_O.

The ^1^H NMR spectrum of PELAVF1S (Figure 4) revealed interesting signals belonging to the main component already detected by HPLC-MS analysis: particularly, at 3.69 ppm, which is the *dd* typical of 3,4-dihydroxyphenylglicole; at 1.11 ppm, which is the CH_3_ of the sugar residue, and at 2.79 ppm, which is the CH_2_ of the ether moiety in verbascoside. Furthermore, certain signals are clearly detected in the following ranges: at 3.49–3.57–3.61 ppm, the sugar signals of the glycoside oleouropein; at 3.54 ppm, the CH-OH of quinic acid; at 3.70 ppm, the CH_2_-O of HT glucoside isomer 1; and between 3.68 and 3.78 ppm, the signals specific to the pectin polymer. The singlet of OH groups are not detected, confirming the ester formation in the polymer pectin with the phenolic counterpart present in the extract.

**Figure 5:**
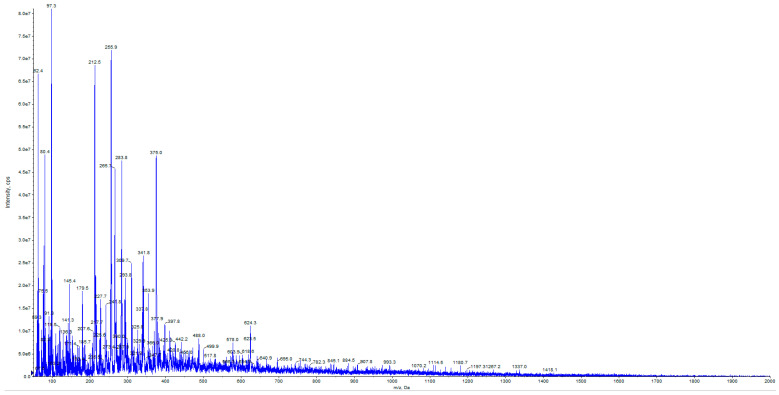
Mass spectrum of PELAVFS1 in negative full-scan mode.

**Figure 6:**
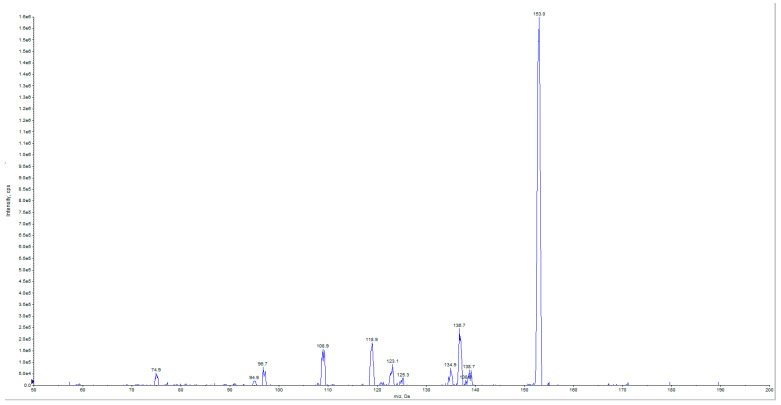
Mass spectrum of HT acquired in negative product ion scan (PIS) mode of PELAVFS1.

As for ELAVFS1, mass spectrometric analysis showed the presence of some important bioactive compounds also in PELAVFS1. The full ion scan spectrum of PELAVFS1 is, in fact, characterized by peaks at *m*/*z* attributable to phenolic compounds (Figure 5). In particular, the species at *m*/*z* 163, 241, and 389 could be attributed to *p*-coumaric acid, elenoic acid, and oleoside, respectively. The species at *m*/*z* 179 and 199 are representative of caffeic acid and the hydroxylated product of the dialdehydic form of decarboxymethyl elenolic acid, respectively. The species at *m*/*z* 257 and 181 indicate the presence of hydroxy oleuropein aglycon. The species at *m*/*z* 357 and 393 could be related to pinoresinol and the 10-hydroxylated product of the dialdehydic form of decarboxymethyl elenolic acid, respectively. The species at *m*/*z* 623 is representative of verbascoside. The species at *m*/*z* 75, 97, 109, 123 and 125 are the main fragment ions of HT and its glucoside, having pseudomolecular ions [M–H]^−^ at *m*/*z* 153 and 315, respectively, but no visibility in the full ion scan spectrum (Figure 5). The presence of HT and HT glucoside, a sugar moiety of hexohesane linked with a unit of HT, was demonstrated by searching the HT precursors. The mass spectrum of HT in product ion scan (PIS) mode is shown in Figure 6. Mass spectrometric analysis was also conducted on commercial pectin, but its full ion scan spectrum did not show the presence of phenols.

### 2.5. Cytotoxicity Evaluation by NRU 

**Figure 7:**
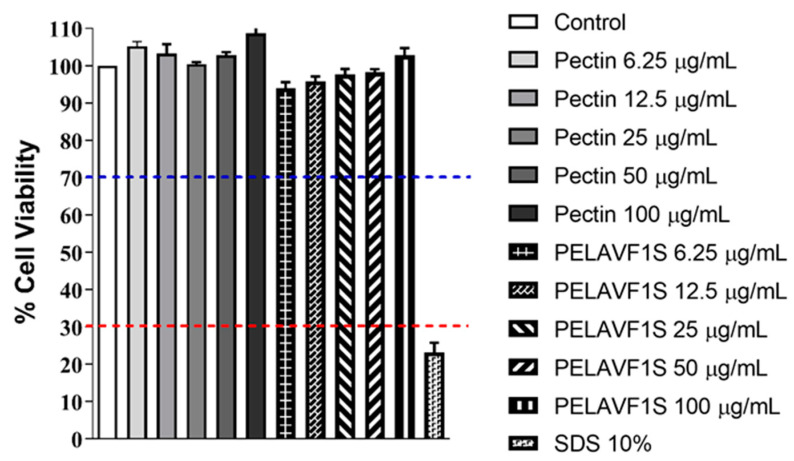
Clone A31 Balb/3T3 fibroblast cell viability NRU test (%) in the absence or in the presence of increasing doses of pectin or PELAVF1S. Each column represents the mean ± SD of 3 wells/group. RED LINE: strongly cytotoxic, cell viability < 30%, BLUE LINE: noncytotoxic, 100 < cell viability < 70%.

The cytotoxic and/or pro-sensitizing effects of pectin or PELAVF1S were evaluated on Balb/3T3 Clone A31 fibroblast cells using the NRU test, according to ISO 10993. The results are shown in Figure 7. The treatment with increasing concentrations of pectin or PELAVF1S did not alter the cell viability compared to the control, indicating the absence of toxic or pro-sensitizing effects of the tested substances.

The quality of cell integrity was assessed after 24 h of incubation using an inverted microscope, while the biological reactivity, including malformations and cellular degeneration, was classified by assigning a score from 0 to 4 reported in ISO 10993-5. The results obtained are shown in Table 4 and Table 5.

The results obtained confirmed the nontoxicity of the examined compounds.

### 2.6. In Vitro Analysis of Pro-Sensitizing Potential (h-CLAT)

The evaluation of the sensitizing effect of our compounds was made on a THP-1 cell line and performed by h-CLAT. The h-CLAT was completed as previously shown by Trombino et al. [24]. The results obtained, treating THP-1 cells with different serial dilutions of pectin and PELAVF1S, are reported in (Table 6) and confirmed that PELAVF1S shows no sensitization effects.

### 2.7. In Vitro Skin Irritation

**Figure 8:**
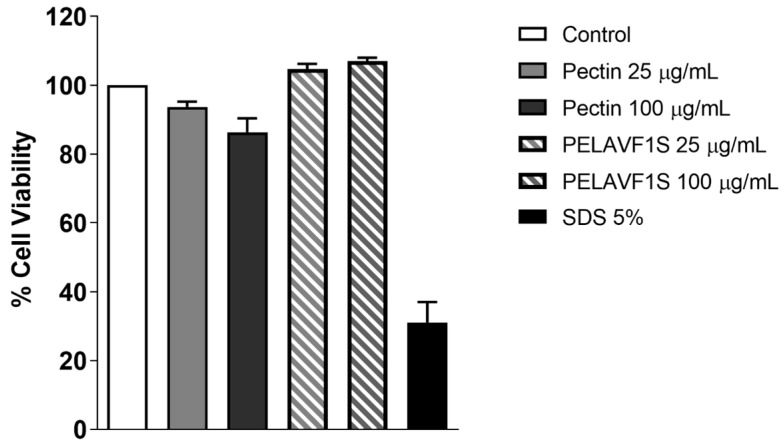
Cell viability in RHE model after treatment with Pectin and PELAVIS. Each substance was tested on RHE tissues reconstructed from three different cell batches. Bars represent mean with SEM.

In vitro skin irritation of PELAVF1S was carried out by the MTT viability assay on the RhE model, as indicated by the Organization for Economic Co-operation and Development guidelines (OECD TG431 and TG439). The MTT evaluates mitochondrial reductase activity, which predicts cell viability. For this purpose, pectin and PELAVF1S were added to the apical side of the EpiDerm™ RhE inserts; these 3D tissues, due to their high sensitivity, are widely used. The results obtained demonstrated that treatment with PELAVIS increases the percentages of cell viability (>50%) compared to the positive control (Figure 8).

### 2.8. Hemolytic Effects of Pectin and PELAVF1S on Peripheral Blood

**Figure 9:**
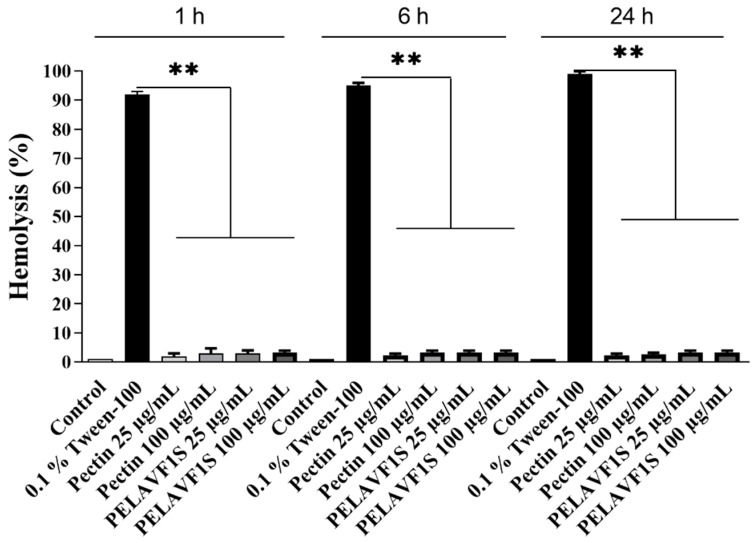
Hemolytic effects of pectin and PELAVF1S: RBCs treated with PBS (control), 0.1% Tween-100 or pectin (25 and 100 µg mL^−1^) or PELAVF1S (25 and 100 µg mL^−1^), for 1 h, 6 h or 24 h. Histograms represent the relative percentage of hemolysis from three different experiments, each performed with triplicate samples. *p* values were calculated against 0.1% Tween-100; ** *p* value < 0.01.

The hemolytic effect of PELAVIF1S was assayed on human blood by means of a hemolysis test. This test is commonly used to evaluate the toxicity of plant extracts of interest in the medical field [25]. The test was performed using peripheral blood of healthy volunteers treated with increasing doses of pectin and PELAVIF1S. The results, shown in Figure 9, highlighted the non-hemolytic characteristic of all the extracts with an acceptable hemolysis rate up to the maximum tested concentration (100 µg mL^−1^).

### 2.9. Proliferative Effects of Pectin and PELAVF1S Extracts on BJ Fibroblast and HaCaT Cells

**Figure 10:**
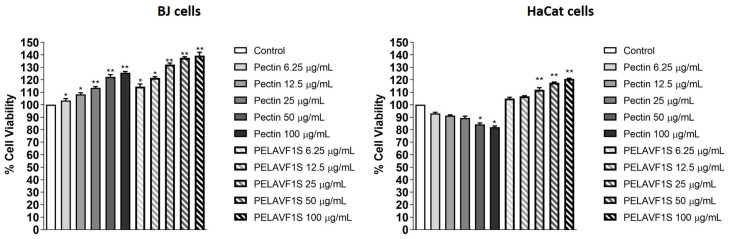
MTT cell proliferation assay. BJ and HaCaT cells treated with increasing doses of pectin and PELAVF1S for 24 h. Results are expressed as a percentage of mean absorbance values compared to the control and represent the mean ± SE of 3 different experiments. * *p* < 0.01; ** *p* < 0.001 compared to control.

The proliferative effects in the experimental models of BJ fibroblast and HaCaT cells were investigated using the anchorage-dependent assay, MTT. The recorded results showed that treatment with increasing doses of PELAVF1S (6.5, 12, 25, 50 and 100 µg mL^−1^) augmented the incorporation of the substrate 3-(4,5-dimethylthiazol-2-yl)-2,5-diphenyltetrazolium (MTT) bromide. These results demonstrate that the compounds are capable of stimulating cell proliferation in a dose-dependent manner in both experimental models (Figure 10).

### 2.10. Effects of Pectin Extracts and PELAVF1S on Cell Motility

**Figure 11:**
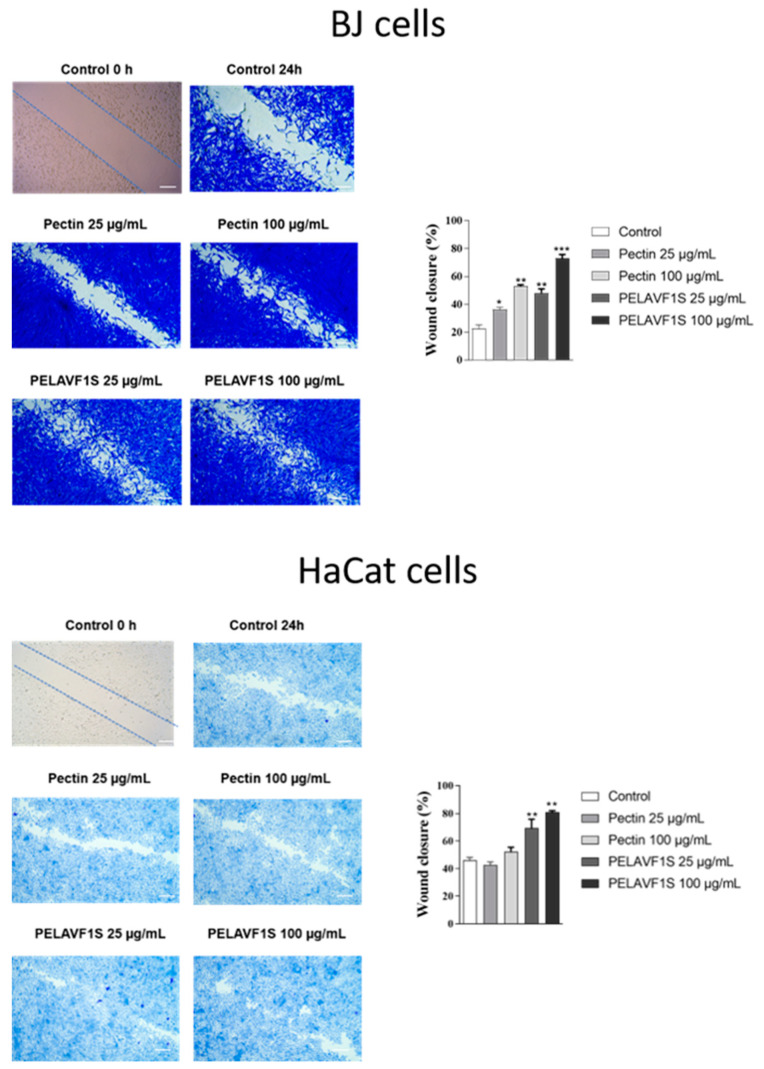
Effects of pectin and PELAVF1S on cells motility. Wound-healing scratch assay in cells treated with pectin (25 and 100 µg mL^−1^) or PELAVF1S (25 and 100 µg mL^−1^). After 24 h of treatment, the cells were stained with Brilliant Blue Coomassie and then photographed under an OLYMPUS BX-51 microscope at 10× magnification. The histogram represents the relative percentage of cut closure, which was calculated by image analysis using ImageJ software (V 1.8.0) Scale bar: 100 μm. * *p* < 0.05; ** *p* < 0.01, *** *p* < 0.001.

The effects on cell motility were evaluated by wound-healing assays. The BJ fibroblast and HaCaT cells were starved in serum-free medium for 24 h. When the confluency was 100%, a scratch was performed, and subsequently, they were treated with pectin (25 and 100 µg/mL) or PELAVF1S (25 and 100 µg mL^−1^). Cell motility was observed under a microscope and photographed after 24 h. The results obtained showed that PELAVF1S-treated cells increased their motility to close the scratch compared to using pectin alone, confirming that the presence of the extract improves the wound-healing activity. The results are shown in Figure 11.

### 2.11. PELAVF1S Increases Lumican Expression in HaCat Cells

**Figure 12:**
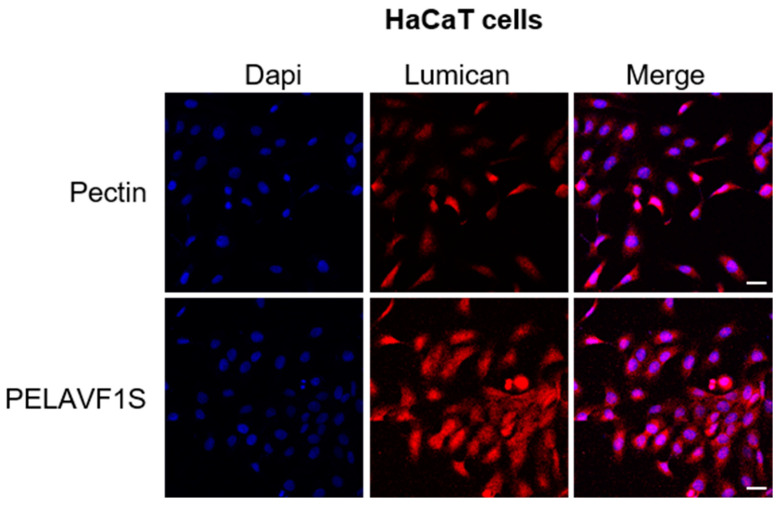
PELAVF1S treatment enhanced Lumican expression in HaCaTcells. Lumican expression was determined by immunofluorescence analysis. DAPI staining was used to visualize the cell nucleus. Scale bars: 25 μm.

Wound healing, responsible for tissue repair, was assessed by lumican determination, which is a small proteoglycan expressed in the extracellular matrix. For this purpose, we investigated whether PELAVF1S (100 µg mL^−1^) was able to stimulate the production of Lumican in HaCaT cells. The results obtained showed a significant increase in Lumican expression after treatment with PELAVIS (Figure 12). 

### 2.12. PELAVF1S Stimulates the Expression of Collagen1 in BJ Fibroblast Cells

**Figure 13:**
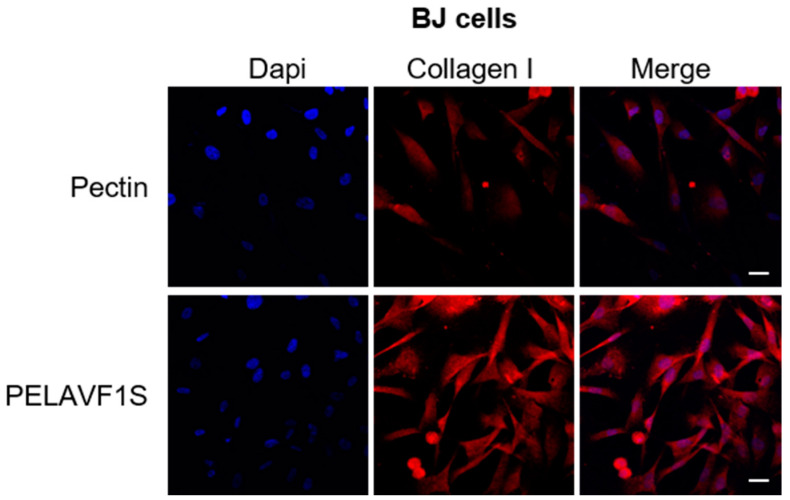
PELAVF1S treatment enhanced collagen type I expression in BJ fibroblast cells. Collagen type I expression was determined by immunofluorescence analysis. DAPI staining was used to visualize the cell nucleus. Scale bars: 25 μm.

Collagen type I is chemotactic for various cells such as fibroblasts, keratinocytes and monocytes. Collagen stimulates the migration of epithelial cells, which is essential for the repair and healing of epidermal wounds [26]. The BJ fibroblast cells were incubated with (100 µg mL^−1^) of PELAVF1S to evaluate the expression of collagen type 1. The results obtained highlighted how PELAVF1S stimulates the production of collagen type I in fibroblasts (Figure 13), helping in wound repair. 

## 3. Materials and Methods

### 3.1. Materials

Tara gum, esterified pectin from citrus fruits with a methoxylation degree of 55–70%, gallic acid (GA), (+)-catechin hydrate (CT), L-ascorbic acid, hydrogen peroxide (H_2_O_2_) at 30%, Folin–Ciocalteu reagent, sodium carbonate (Na_2_CO_3_), 2,2′-diphenyl-1-radical picrylhydrazyl (DPPH), 2,2′-azino-bis(3-ethylbenzothiazolin-6-sulfonic) radical (ABTS), potassium persulfate (K_2_S_2_O_8_), ammonium molybdate tetrahydrate (NH_2_)_2_MoO_4_), sodium molybdate (Na_2_MoO_4_), sodium nitrite (NaNO_2_), sodium phosphate (Na_3_PO_4_), aluminum chloride (AlCl_3_), hydrochloric acid (HCl), sodium hydroxide (NaOH), acid sulfuric acid (H_2_SO_4_ 96% *w*/*w*), absolute ethanol, methanol, analytical acetone, *n*-hexane, ethyl acetate, dichloromethane, Whatman No. 3 filter paper, dialysis membrane (MWCO: 10,000 Da), DMSO, CDCl_3_, and D_2_O were purchased from Sigma Aldrich (Sigma Chemical Co., St Louis, MO, USA). LC-MS grade water, acetonitrile, formic acid, and HT were supplied from Merk Life Science S.r.l. (Milano, Italy). Methanol was purchased from Sigma-Aldrich (Riedel-de Haën, Laborchemikalien, Seelze, Germany).

Eagle’s Minimum Essential Medium, Trypsin, Penicillin/Streptomycin, DMEM High Glucose, HEPES, CBS, β-mercaptoethanol, MTT, DMSO, SDS, Neutral Red, NiSO_4_, FACS Buffer and Propidium Iodide (PI) were purchased from Sigma Aldrich, Milan, Italy. RPMI 1640, phosphate-buffered saline (PBS), fetal bovine serum (FBS), and CD86 were purchased from Life Technologies (Monza, Italy), while sodium pyruvate was purchased from Gibco, UK and CD54 was purchased from (Invitrogen, Carlsbad, CA, USA). 

### 3.2. Instruments

Centrifugation techniques were performed using the Thermo Electron Corporation ALC Multispeed Centrifuge. The drying process was carried out using the Micro Modulyo freeze dryer provided by Edwards. The absorbance values of the samples were obtained using the Jasco V-530 UV-vis spectrophotometer (Jasco Inc., Easton, MD, USA). The operations of evaporation of the solvent, during the phase of extraction, were performed by a BUCHI rotary evaporator. HPLC analyses were performed using an HPLC 1100 system (Agilent Technologies, Palo Alto, CA, USA) equipped with a binary pump, a thermostat column compartment, an autosampler, and a variable wavelength UV detector (VWD); then, they were interfaced to a triple–quadruple hybrid mass detector (QQQ 6430, Agilent technologies). Pectin and PELAVF1S were qualitatively characterized by electrospray ionization tandem mass spectrometry (ESI-MS/MS) using an API 4000 Q-Trap mass spectrometer (MSD Sciex Applied Biosystem, Foster City, CA, USA) in negative ion mode. NMR spectra were acquired with a Bruker *Advance 200* (300 mHz for ^1^H and 75 MHz for ^13^C) and processed by XWin-NMR.

### 3.3. Samples Preparation

#### Extraction Procedure

The olive mill wastewater (OMW) employed in this work was offered by the Company Vinciprova Srl of San Vincenzo la Costa (CS) Italy during the 2019 oil season. This waste is from *Olea Europea Roggianella cv*; it was harvested in October and immediately processed using the traditional method Enorossi 150 working at 150 kg of olives. Several samples of 50 mL each were stored at −50 °C before the analysis. OMW (200 mL) has been filtered thought Whatman paper No. 3 and centrifugated (3 times) for 10 min at 10,000 rpm. The liquid phase was freeze-dried and furnished as a brown dry solid (ELAVF) that was stored at +4 °C until using. The extracts were obtained via two methods: maceration, (0.5 g of ELAFV) employing ethanol, acetone, *n*-hexane, and dichloromethane as solvents (40 mL), and Soxhlet, (1 g of ELAVF) at reflux for 5 h, employing ethanol, acetone, dichloromethane, and ethanol/water (90:10) as solvents (100 mL). After the extraction, all the extracts (ELAVF) were filtered on Whatman paper No. 3, freeze dried and stored at +4 °C until the analysis.

### 3.4. Extracts Characterization

#### 3.4.1. Colorimetric Assays

The extracts were analyzed by the evaluation of total phenolic, phenolic acid, flavonoid, and anthocyanin concentrations, whereas their antioxidant performances were evaluated by scavenger tests against hydrophilic and lipophilic radical species.

##### Total Phenolic Content Determination

The total phenolic content (TPC) was evaluated by the Folin–Ciocalteu assay following a procedure found in the literature with some modifications [27] as reported in the SI section. The TPC values of each extract were expressed as weight of CT per gram of sample (mg CT/g sample).

##### Total Phenolic Acid Determination

For the evaluation of the total content of phenolic acids (PAC), the Arnov test with some changes was used [28] (see the SI section for details). The PAC values were expressed as the weight of CT per gram of sample (mg CT/g sample) after having carried out the relative calibration line. 

##### Flavonoid Content Determination

The total flavonoid content (FC) of each extract was evaluated by a method reported in the literature with some modifications [27] (see the SI section for details). The FC values were expressed as the weight of CT per gram of sample (mg CT/g sample) after carrying out a suitable calibration curve.

##### Total Antioxidant Capacity

A literature protocol with a few changes was employed to determine the total antioxidant capacity (TAC) of each extract [29]. The total antioxidant activity of each matrix was expressed as the CT equivalent concentration (mg CT/g sample).

##### Scavenger Activity against DPPH Radical

The scavenging activities in the organic environment were evaluated in terms of reduction of the radical 2,2′-diphenyl-1-picrilhydrazyl (DPPH), using the procedure re-ported in the literature with some changes [30] (see the SI section for details). The scavenging activity on the lipophilic DPPH radical was expressed in terms of IC_50._

##### Scavenger Activity against ABTS Radical

The scavenging activities in the aqueous medium were determined in terms of reduction of the radical species 2,2′-azino-bis(3-ethylbenzothiazolin-6-sulphonic) (ABTS), as reported in the literature with some changes [31] (see the SI section for details). The scavenging activity of the analyzed system was expressed in terms of IC_50_.

#### 3.4.2. HPLC-MS/MS Analysis of ELAVF 

The chromatographic separation of polyphenols in the ELAVF extracts was conducted by using an HPLC 1100 system equipped with a degasser, quaternary pump solvent delivery, thermostatic column compartment, autosampler, single wavelength UV-Vis detector, and MSD triple quadrupole QQQ 6430 in a series configuration (Agilent Technologies, Palo Alto, CA, USA). Specifically, the lyophilized ELAVFs were resuspended in 2 mL of the extraction solvent to a final concentration of ~1.2 mg mL^−1^ and filtered through 0.2 μm pore-size regenerated cellulose filters (VWR International Srl, Milano, Italy). Then, 3 μL of each sample (analyzed in triplicate) was injected into a reversed stationary phase column, Luna C_18_ (150 × 2 mm i.d., particle size 3 μm, Phenomenex, Torrance, CA, USA) protected by a C_18_ Guard Cartridge (4.0 × 2.0 mm i.d., Phenomenex), and a binary mobile phase composed of (solvent A) H_2_O/formic acid 0.1% (*v*/*v*) and (solvent B) acetonitrile (Chromasolv, VWR International Srl, Milano, Italy) was employed with the following gradient: 0 min, 10% B; 1 min, 10% B; 15 min, 30% B; 22 min, 50% B; 28 min, 100% B; 34 min, 100% B; 36 min, 10% B. The column temperature was controlled at 20 °C, and the flow was maintained at 0.4 mL min^−1^. The UV-Vis detection wavelength was set at 280 nm.

Mass Hunter Workstation software (version B.01.04; Agilent Technologies) was employed to acquire and process MS and MS/MS data in negative ionization mode (*m*/*z* 50–1200) by setting the capillary voltage at 4000 V and nitrogen as drying (T = 350 °C; flow rate = 9 L min^−1^) and nebulizing gas (40 psi). Compound identification was achieved by matching different information, such as UV absorption, retention times (RTs), elution order, and mass spectra (MS and MS/MS), with those already reported in the literature [20,32]. The revealed compounds were quantified by multiple reaction monitoring (MRM), and their concentrations were expressed as μg mL^−1^ of HT equivalents (concentration range 0.01125–10 μg mL^−1^; R^2^ = 0.99923).

#### 3.4.3. NMR Analysis

Each sample (20 mg) was dissolved in 600 µL of DMSO, and the NMR spectra were acquired with a Bruker *Advance 200* (300 mHz for ^1^H and 75 MHz for ^13^C) and processed by XWin-NMR in the following conditions: temperature 25 °C, NS 64, D1 2.00000000 s, using the mode water suppression. 

### 3.5. Pectin Polymer Synthesis (PELAVF1S)

For the synthesis of PELAVF1S, a known procedure [33] with some modifications was exploited. The amount of ELAVF1S equivalent to 4.7 mg of catechin was solubilized in distilled water (30 mL) and mixed with a pectin solution (62 mg in 30 mL of distilled water) and 1 g of pancreatic porcine lipase (PPL). The mixture was stirred and heated at 50 °C for 24 h. After this, 60 mL of dry ethanol was added to foster the ELAVF1S solubilization and the solid polymer separation. The mixture was centrifuged at 4000 rpm for 5 min (three times). The solid was recovered and added with 60 mL of distilled water; then, it was stirred at room temperature for 10 min to isolate the PPL not linked (solid) from the pectin polymer. The suspension was centrifuged at 4000 rpm for 5 min, and the supernatant was freeze dried, furnishing a billowing white conjugated pectin polymer.

### 3.6. Pectin Polymer ESI-MS/MS Characterization (PELAVF1S)

MS characterization was performed by electrospray ionization tandem mass spectrometry (ESI-MS/MS) in negative ion mode because of the polar nature of phenolic compounds. More specifically, direct infusion analysis (FIA) was used to optimize the instrumental parameters and maximize the clarity and the readability of the spectra. In particular, the instrumental parameters were as follows: entrance potential (EP), −14 eV; declustering potential (DP), −70 eV; collision energy (CE) and collision exit potential (CXP), −25 and −10 eV, respectively. Analyses in full scan, product and precursor ion scan, and neutral loss have been performed to investigate the presence of phenols linked to pectin in the synthetized polymer.

The dry matrices were dissolved in a solution of water/methanol (*v*/*v* 80:20), filtered through a 0.45 µm PVDF filter (Merk, Darmstadt, Germany) and analyzed by mass spectrometry.

### 3.7. Cell Lines and Culture Conditions

The BJ (human fibroblast), Balb/3T3 Clone A31 (murine fibroblast), HaCaT (immortalized human keratinocytes) and THP-1 (human monocyte) cell lines were purchased from ATCC, Manassas, VA, USA. BJ and HaCaT cells were cultured in Eagle’s Minimum Essential Medium supplemented with 10% FBS and 1% Penicillin/Streptomycin. Balb/c 3T3 clone A31 cells were maintained in DMEM with 10% CBS and 1% Penicillin–Streptomycin, while THP-1 cells were cultured in RPMI 1640 medium with 10% FBS, 1% Penicillin/Streptomycin and 0.05% β-Mercapto-ethanol. All cell lines were maintained at 37 °C in modified air containing with 5% humidified CO_2_.

### 3.8. Neutral Red Uptake Assay (NRU)

The NRU test (ISO 10993-5:2009 “Biological evaluation of medical Devices-Part 5: Tests for in vitro cytotoxicity” [34]) was performed on Balb/3T3 Clone A31 cells. Cells (2.5 × 10^4^) were treated with increasing doses of pectin (6.5, 12, 25, 50, and 100 µg mL^−1^) or PELAVF1S (6.5, 12, 25, 50, and 100 µg/mL) in DMEM for 24 h at 37 °C and 5% CO_2_ atmosphere. Cell viability was tested by a neutral red uptake (NRU) assay, which included incubation (3 h) with a neutral red solution (50 µg/mL) and was followed by extraction with acetic acid, ethanol and water (1:50:49 *v*/*v*/*v*) [35]. Absorbance was measured at 540 nm using an Epoch microplate reader (BioTek, Winooski, VT, USA). The percentage of viability was calculated as follows:

%Viability = [Abs (540 nm)_test material_ − Abs (540 nm)_blank_]/[Abs (540 nm)_control_ − Abs (540 nm)_blank_]

### 3.9. Human Cell Line Activation Test (h-CLAT) 

h-CLAT aims to evaluate whether substances or mixtures cause activation of the immune system, resulting in skin sensitization according to the method described by the Organization for Economic Co-operation and Development (OECD) 442E [24] and in the EURL 158 protocol ECVAM (European Union Reference Laboratory for Alternatives to Animal Testing). The test was performed on THP-1 cells, evaluating the modulation of the expression of two costimulatory molecules, CD54 and CD86, using nickel sulfate (NiSO_4_) as a positive control. An increased expression of CD54 and CD86 on monocytes correlates with the activation of an immune response following exposure to a partially allergenic antigen. THP-1 cells were cultured in RPMI 1640 medium with 10% FBS, 1% Penicillin/Streptomycin and 0.05% mM β-Mercaptoethanol and then plated in a 96-well multi-well at a concentration of 1.5 × 10^5^ cells per well. After 24 h of incubation, the cells were centrifuged and the treatments were added. The next day, the samples were centrifuged and resuspended in FACS buffer in the presence of PI (propidium iodide); then, by flow cytometry, the CV75, i.e., the concentration causing 25% of mortality, was calculated for each tested substance from subsequent use for the actual test, as described in Test No. 442E: In Vitro Skin Sensitization [36]. NiSO_4_ (100 μg mL^−1^) was used as a positive control, while the culture medium was used as a negative control. The experiment was repeated on 3 different days and performed in 3 replicates. After incubation with the treatments, the cells were centrifuged and re-suspended in FACS buffer and then divided into three aliquots. They were then centrifuged, re-suspended in blocking solution (FACS buffer containing 0.01% γ globulin) and subsequently incubated for 15 min at 4 °C. Finally, the cells were stained with a fluorescein antibody targeting CD86, CD54 or IgG1, with the latter used as a control, for 30 min at 4 °C. FACS buffer washes were performed, and an additional FACS buffer was added with PI. The expressions of CD54, CD86 and cell viability levels were then evaluated by flow cytometry, and the results were calculated as previously described [37].

### 3.10. In Vitro Skin Irritation OECD 439

Skin irritation caused by chemical compounds was assessed using the reconstructed human epidermis test method (OECD 439) [38]. The reconstructed human epidermal Epi-Derm™ (RhE) plate was activated overnight in a humidified incubator at 37 °C and 5% CO_2_. Then, 100 μg of PELAVF1S was applied on top of the RhE, and its effect was compared with that obtained from sodium dodecyl sulfate SDS (5%) and PBS, which were used as positive and negative controls, respectively. After 15 min of treatment, tissues were rinsed with PBS, transferred to 2 mL of fresh medium, and incubated for 42 h. Subsequently, tissue viability was assessed using the MTT test, as previously described [39]. All tests were performed three times. Cell viability was expressed as a percentage and calculated using the following equation:

Viability (%) = (OD sample × 100)/OD negative control

### 3.11. Hemolysis Assay

Fresh human blood from healthy volunteers was collected in sodium citrate tubes and centrifuged at 2000 rpm for 10 min to isolate red blood cells (RBCs) as a pellet, as previously described [37]. RBCs were washed three times with cold PBS pH 7.4 and re-suspended in the same buffer (10% *v*/*v*). Subsequently, pectin (25 and 100 µg mL^−1^) or PELAVF1S (25 and 100 µg mL^−1^) were added to the erythrocyte suspension and incubated for 24 h at 37 °C. Hemoglobin release was determined after centrifugation (2000 rpm, 10 min) by photometric analysis of the supernatant at 540 nm at different endpoints (1, 6 and 24 h), using a microplate reader (Synergy H1 microplate reader, BioTek). Complete hemolysis was achieved using 0.1% (*v*/*v*) Triton X-100, which produced the 100% positive control value, while PBS provided the negative control value. The study procedure with human blood was approved by the Ethics Committee of the University of Calabria (Unical AOO1 Amministrazione Centrale, Doc. No. 234 dated 14 January 2021).

### 3.12. Cell Viability Assay

Cell viability was evaluated by the 3-(4,5-dimethylthiazol-2-yl)-2,5-diphenyltetrazolium (MTT) assay. BJ fibroblast and HaCaT cells (4 × 10^4^) were 48 multi-well plated and synchronized in serum-free media (SFM) for 12 h. Subsequently, they were treated with increasing doses of pectin or PELAVF1S (6.5, 12, 25, 50 and 100 µg/mL). After 24 h of treatment, 200 µL of MTT (5 mg/mL) was added for 2 h at 37 °C. Finally, 200 µL of DMSO was added to each well, and the optical density was measured at 570 nm using a Beckman Coulter microplate reader [33]. For each sample, eight replicates were performed.

### 3.13. Wound-Healing Scratch Assay

BJ fibroblast cells and HaCaT cells were grown to confluence in regular media and then maintained in SFM for 12 h. The monolayers were scratched as previously described [33] and treated with pectin (25 and 100 µg/mL) or PELAVF1S (25 and 100 µg/mL). Then, wound healing was photographed at 24 h at x4 magnification using phase-contrast microscopy (CKX-53 Olympus).

### 3.14. Immunofluorescence

HaCaT and BJ cells were cultured on glass coverslips and treated with 100 μm of PELAVF1S for 24 h, washed with PBS, and then fixed with 4% paraformaldehyde in PBS for 20 min at room temperature. After permeabilization with 0.2% Triton X-100 in PBS for 5 min, the cells were blocked with 5% bovine serum albumin for 30 min and incubated overnight with Lumican (1:200) or anti-collagen I antibody (1:250) in PBS overnight at 4 °C. Then, the cells were washed three times with PBS and incubated with the secondary antibody anti-mouse IgG–fluorescein isothiocyanate (1:200) for 1 h at room temperature. To check the specificity of immunolabeling, the primary antibody was replaced by normal mouse serum (negative control). Immunofluorescence analysis was carried out on a OLYMPUS FV3000 microscope using a ×40 objective.

### 3.15. Statistical Analysis

The inhibitory concentration 50 (IC50) was evaluated by nonlinear regression performed with Prism GraphPad Prism, version 4.0 for Windows (GraphPad software). A one-way analysis of variance (ANOVA) was performed on the samples and subsequently a multi-comparison Dunnett’s test. In vitro data were analyzed by Student’s *t*-test using the GraphPad Prism 8.3.0 (GraphPadSoftware, Inc., San Diego, CA, USA). *p* < 0.05 was considered statistically significant.

## 4. Conclusions

The conjugation of natural extracts to a pectin biopolymer is a good technology to build innovative tools that are useful for non-healing chronic wounds. Ethanolic extract of OMW rich in valuable secondary metabolites, mainly HT, was successfully linked by an enzymatic transesterification using porcine pancreatic lipase, as demonstrated by ESI MS/MS spectra, to obtain a functional polymer able to promote proliferative fibroblasts activity in an in vitro scratch assay and showing a safe profile, thanks to the absence of sensitization and hemolysis. Furthermore, treatment with PELAVF1S on fibroblast (BJ cells) and keratinocytes (HaCaT cells) showed a proliferative and healing effect, as demonstrated by cell viability and motility assays. Our results highlighted how PELAVF1S stimulates in HaCaT cells the Lumican expression, which is a small leucine-rich proteoglycan expressed in the extracellular matrices of several tissues. Lumican regulates collagen fibrillogenesis and keratinocyte phenotypes and appears to be involved in inflammatory cell extravasation and angiogenesis, both being central in the wound-healing process [40]. The results obtained demonstrated that the treatment with PELAVF1S increased the expression of Lumican. Furthermore, it was observed that PELAVF1S stimulated the collagen production in the BJ fibroblast cells, confirming the healing and proliferative effects of PELAVF1S in the dermal compartment. This research demonstrated how waste can be a resource to be exploited as a raw material to obtain a functional biopolymer to be used as an active ingredient in a wide range of topical wound-healing drug formulations.

## Figures and Tables

**Table 1:** Extractions of OMW from Olea Europea *Roggianella cv*.

Sample		Solvent	Volume (mL)	Condition		Yield	
Code	Mass (g)			T (°C)	*t* (h)	(g)	(%)
**ELAVF1S**	1.0	Ethanol	100	60	5	0.2560	25.60
**ELAVF1M**	0.5		40	r. t.	96	0.0830	8.30
**ELAVF2S**	1.0	Acetone	100	60	5	0.0510	5.10
**ELAVF2M**	0.5		40	r. t.	96	0.0007	0.07
**ELAVF3S**	1.0	Dichloromethane	100	60	5	0.0290	2.90
**ELAVF3M**	0.5		40	r. t.	96	0.0120	1.20
**ELAVF4S**	1.0	*n*-hexane	100	60	5	0.0100	1.10
**ELAVF4M**	0.5		40	r. t.	96	0.0090	0.90
**ELAVF5S**	1.0	Ethanol/water 90/10 (*v*/*v*)	100	60	5	0.5210	52.10
**ELAVF5M**	0.5		40	r. t.	96	0.0090	0.90

ELAVF1S = olive mill wastewater ethanol extract Soxhlet; ELAVF1M = olive mill wastewater ethanol extract maceration; ELAVF2S = olive mill wastewater acetone extract Soxhlet; ELAVF2M = olive mill wastewater acetone extract maceration; ELAVF3S = olive mill wastewater dichloromethane extract Soxhlet; ELAVF3M = olive mill wastewater dichloromethane extract maceration; ELAVF4S = olive mill wastewater *n*-hexane extract Soxhlet; ELAVF4M = olive mill wastewater *n*-hexane extract maceration; ELAVF5S = olive mill wastewater ethanol/water 90/10 (*v*/*v*) extract Soxhlet; ELAVF5M = olive mill wastewater ethanol/water 90/10 (*v*/*v*) extract Soxhlet maceration. r. t. = room temperature.

**Table 2:** Antioxidant characterization of LOMW extracts.

Sample	TPC (mg CT/g)	PAC (mg CT/g)	FL (mg CT/g)	TAC (mg CT/g)	IC_50_ (mg mL^−1^)	
Code					DPPH Radical	ABTS Radical
**LAVF**	75.50 ± 2.71	50.82 ± 1.24	34.08 ± 1.15	1.105 ± 0.051	0.095 ± 0.003	0.0185 ± 0.0007
**ELAVF1S**	94.16 ± 3.52 ^a^	65.92 ± 1.94 ^a^	62.22 ± 2.1 ^a^	1.023 ± 0.023 ^a^	0.152 ± 0.005 ^a^	0.0371 ± 0.0012 ^a^
**ELAVF1M**	60.00 ± 2.21 ^b^	40.21 ± 1.25 ^b^	40.21 ± 1.25 ^b^	0.651 ± 0.022 ^b^	0.245 ± 0.010 ^b^	0.0574 ± 0.0021 ^b^
**ELAVF2S**	28.98 ± 0.97 ^c^	20.75 ± 0.73 ^c^	18.62 ± 0.73 ^c^	0.309 ± 0.009 ^c^	0.485 ± 0.021 ^c^	0.1202 ± 0.0043 ^c^
**ELAVF2M**	24.18 ± 0.88 ^d^	15.90 ± 0.45 ^d^	15.51 ± 0.45 ^d^	0.262 ± 0.008 ^d^	0.599 ± 0.022 ^d^	0.1458 ± 0.051 ^d^
**ELAVF3S**	1.03 ± 0.03 ^h^	0.72 ± 0.02 ^h^	0.68 ± 0.02 ^h^	0.019 ± 0.001 ^g^	3.935 ± 0.174 ^h^	2.048 ± 0.0845 ^i^
**ELAVF3M**	2.59 ± 0.11 ^f^	1.63 ± 0.05 ^f^	1.54 ± 0.05 ^f^	0.034 ± 0.001 ^f^	1.725 ± 0.074 ^f^	1.389 ± 0.0428 ^g^
**ELAVF4S**	1.39 ± 0.05 ^g^	0.98 ± 0.03 ^g^	0.98 ± 0.03 ^g^	0.018 ± 0.001 ^g,h^	2.052 ± 0.095 ^g^	1.789 ± 0.0528 ^h^
**ELAVF4M**	0.66 ± 0.02 ^i^	0.39 ± 0.01 ^i^	0.31 ± 0.01 ^i^	0.017 ± 0.001 ^h^	4.351 ± 0.141 ^i^	3.4862 ± 0.1415 ^j^
**ELAVF5S**	22.93 ± 0.79 ^d^	15.97 ± 0.40 ^d^	15.10 ± 0.40 ^d^	0.257 ± 0.007 ^d^	0.620 ± 0.017 ^d^	0.2581 ± 0.0098 ^e^
**ELAVF5M**	9.66 ± 0.22 ^e^	5.76 ± 0.23 ^e^	5.79 ± 0.23 ^e^	0.108 ± 0.003 ^e^	1.028 ± 0.025 ^e^	0.733 ± 0.0254 ^f^

LAVF = lyophilizate olive mill wastewater; ELAVF1S = olive mill wastewater ethanol extract Soxhlet; ELAVF1M = olive mill wastewater ethanol extract maceration; ELAVF2S = olive mill wastewater acetone extract Soxhlet; ELAVF2M = olive mill wastewater acetone extract maceration; ELAVF3S = olive mill wastewater dichloromethane extract Soxhlet; ELAVF3M = olive mill wastewater dichloromethane extract maceration; ELAVF4S = olive mill wastewater *n*-hexane extract Soxhlet; ELAVF4M = olive mill wastewater *n*-hexane extract maceration; ELAVF5S = olive mill wastewater ethanol/water 90/10 (*v*/*v*) extract Soxhlet; ELAVF5M = olive mill wastewater ethanol/water 90/10 (*v*/*v*) extract Soxhlet maceration. TPC = total phenolic content; PAC = phenolic acid content; FC = flavonoid content; TAC = total antioxidant activity; DPPH = 2,2’-diphenyl-1-picrilhydrazyl; ABTS = 2,2’-azino-bis(3-ethylbenzothiazolin-6-sulphonic). Data represent mean ± RSD (*n* = 3). Different letters in the same column express significant differences (*p* < 0.05).

**Table 3:** HPLC-MS/MS acquisition parameters and phenolic compound content in ELAVF extracts.

Compound	RT (min)	[M–H]^−^ (*m*/*z*)	MS/MS Fragments	Q1 (*m*/*z*)	Q3 (*m*/*z*)	Frag (V)	CE (V)	ELAVF1S (mg mL^−1^)
Verbascoside residue	1.475	477.1	458.9 (100); 160.8 (21); 151.1 (20); 113.2 (17)	477	459	100	15	1.62 ± 0.15
3,4-dihydroxyphenylglycol	1.515	169.2	150.8 (27); 123.0 (100)	169	123	100	15	0.47 ± 0.04
Quinic acid	1.773	191.0	109.0 (7); 92.8 (68); 85.0 (100); 59.1 (23)	191	85	100	30	4.1 ± 0.4
Hydroxytyrosol glucoside isomer 1	2.245	315.2	153.0 (100); 123.0 (11)	315	153	80	15	21.2 ± 1.9
Hydroxytyrosol glucoside isomer 2	2.470	315.2	153.0 (100); 123.0 (11)	315	153	80	15	12.1 ± 1.1
Hydroxytyrosol	2.957	153.1	123.0 (100)	153	123	100	15	7.1 ± 0.6
Decarboxymethyl-elenolic acid derivative	3.542	185.0	111.1 (100); 94.9 (51); 69.1 (17); 59.0 (24)	185	111	100	15	1.43 ± 0.13
Hydroxylated product of dialdehydic form of decarboxymethyl elenolic acid	4.428	199.3	111.0 (100); 95.1 (23); 85.1 (22); 69.0 (28); 59.1 (72)	199	111	100	15	3.8 ± 0.3
Caffeic acid	7.996	179.1	134.9 (100)	179	135	100	15	6.4 ± 0.6
Decarboxymethyl-elenolic acid (HyEDA)	8.075	183.0	139.0 (100); 108.9 (83); 94.9 (25)	183	139	100	15	0.42 ± 0.04
Oleuropein aglycone derivative	8.124	377.2	197.0 (100); 153 (44)	377	197	80	15	5.6 ± 0.5
*p*-Coumaric acid	10.798	163.1	119.0 (100)	163	119	100	15	5.9 ± 0.3

RT, retention time; Q1, parent ion mass; Q3, daughter ion mass; Frag, fragmentor voltage; CE, collision energy. ELAVF1S: Soxhlet extract in ethanol.

**Table 4:** Results interpretation.

Score	Reactivity	Condition of All Cultures
0	None	No alterations
1	Slight	Presence of some altered cells under the sample
2	Mild	Alteration present in a limited area under the sample
3	Moderate	Alteration present in extending area under the sample up to 1.0 cm
4	Severe	Area extending more than 1.0 cm outside the sample

**Table 5:** Assessment of biological reactivity.

Sample	Biological Reactivity
Control	0
Pectin 25 µg/mL	0
Pectin 100 µg/mL	0
PELAVF1S 25 µg/mL	0
PELAVF1S 100 µg/mL	0
Control + (SDS 10%)	4

**Table 6:** RFI% value of CD54 and CD86 on THP-1 monocyte.

Samples	CD54 *	CD86 *
Pectin 25 µg/mL	60.19	71.15
Pectin 100 µg/mL	62.71	83.28
PELAVF1S 25 µg/mL	61.29	78.95
PELAVF1S 100 µg/mL	65.14	82.49
Control	57.03	64.37
Control + (NISO_4_)	223.05	198.61

* The compound is a skin sensitizer where CD86 > 150 and CD54 > 200.

## Supplementary Materials

The following supporting information can be downloaded at: https://www.mdpi.com/article/10.3390/ijms242015075/s1.
