# Supplementary material for: Synthesis and Characterization of a Biopolymer Pectin/Ethanolic Extract from Olive Mill Wastewater: In Vitro Safety and Efficacy Tests on Skin Wound Healing
Source: Int J Mol Sci. 2023 Oct 11;24(20):15075. doi: 10.3390/ijms242015075 (PMC10606433; doi:10.3390/ijms242015075)
Supplement: Supplementary file 1 [file ijms-24-15075-s001.zip › ijms-2496779-supplementary.pdf]

SUPPLEMENTARY MATERIALS

Synthesis and Characterization of a Biopolymer Pectin/Ethanolic Extract from Olive Mill Wastewater: In Vitro Safety and Efficacy Tests on Skin Wound Healing

Francesca Aiello [1,*], Rocco Malivindi [1], Marisa Francesca Motta [1], Pasquale Crupi [2], Rosa Nicoletti [3,4], Cinzia Benincasa [3], Maria Lisa Clodoveo [2], Vittoria Rago [1], Umile Gianfranco Spizzirri [1,5,†] and Donatella Restuccia [1,†]

[1] Department of Pharmacy, Health and Nutritional Sciences, University of Calabria, 87036 Rende, Italy; rocco.malivindi@unical.it (R.M.); mottamarisaf@icloud.com (M.F.M.); vittoria.rago@unical.it (V.R.); g.spizzirri@unical.it (U.G.S.); donatella.restuccia@unical.it (D.R.)
[2] Interdisciplinary Department of Medicine, University Aldo Moro Bari, 70121 Bari, Italy; pasquale.crupi@uniba.it (P.C.); marialisa.clodoveo@uniba.it (M.L.C.)
[3] Council for Agricultural Research and Economics (CREA), Research Centre for Olive, Fruit and Citrus Crops, Via Settimio Severo 83, 87036 Rende, Italy; rosa.nicoletti@crea.gov.it (R.N.); cinzia.benincasa@crea.gov.it (C.B.)
[4] DICEM—Department of European and Mediterranean Cultures: Architecture, Environment, and Cultural Heritage, University of Basilicata, Matera, Via Lanera, 20, 75100 Matera, Italy
[5] Ionian Department of Law, Economics and Environment, University of Bari Aldo Moro, 74123 Taranto, Italy
* Correspondence: francesca.aiello@unical.it; Tel.: +39-0984-493154
† These authors contributed equally to this work.

Table of Contents

S1. Disposable phenolic groups by Folin–Ciocalteu procedure

S2. Total phenolic acid determination

S3. Flavonoid content determination

S4. Determination of total antioxidant activity

S5. Determination of scavenging activity on DPPH radicals

S6. Determination of scavenging effect on the ABTS radical cation

S1. Total phenolic content determination

For each extract, an aqueous solution at different concentrations of sample were prepared, and 1 mL of Folin-Ciocalteu reagent was added to 6 mL of solution, and, after 3 minutes, 3 mL of aqueous

solution of Na_2CO_3 (2% w/v). The solutions thus prepared were kept under stirring in the dark for 2 h. The absorbance of the samples was then spectrophotometrically measured at the wavelength of 760 nm (Evolution 201 spectrophotometer (Thermo Fisher Scientific, Hillsboro, OR, USA)), against a control solution prepared under the same conditions, but containing 6 mL of purified water. The amount of available phenolic groups was expressed as milligrams of catechin per gram of sample (mg CT/g sample). A calibration curve was constructed, using aqueous solutions of (+)-catechin at different concentrations (8.0, 16.0, 24.0, 32.0, and 40.0 µM). To define the regression line and the R^2 coefficient using the least squares method, the concentrations of the aqueous solutions of CT were correlated to the respective absorbance values.

S2. Total phenolic acid determination

At 1 mL of an aqueous solutions of each sample were added 5.0 mL of purified water, 1.0 mL of HCl 0.5 mol L^{-1}, 1.0 mL of the Arnov's reagent (10.0 g of sodium molybdate and 10.0 g of sodium nitrite were solubilized in 100 mL of purified water) and 1.0 mL of NaOH (4.0% w/v), obtaining a final volume of 10 mL with purified water. A control solution was also prepared under the same conditions by replacing sample solution with purified water. The absorbance of the samples was spectrophotometrically at 490 nm (Evolution 201 spectrophotometer (Thermo Fisher Scientific, Hillsboro, OR, USA)).

S3. Flavonoid content determination

Aqueous solutions of the carob pod extract, gelatin conjugate and gummy extracts were prepared, at different concentrations, and, to 0.5 mL of each, 2.0 mL of purified water and 0.15 mL of $NaNO_2$ aqueous solution (15% w/v) were added. After 6 minutes, 0.15 mL of $AlCl_3$ solution (10% w/v) was added to the stock solution. Subsequently, after 6 minutes, 3.0 mL of NaOH solution (4% w/v) was added, obtaining a final volume of 5 mL with purified water. The solution thus prepared was kept away from light for 15 minutes. A control solution was also prepared under the same conditions but

without sample. The absorbance of the solutions was then measured with a spectrophotometer at a wavelength of 510 nm (Evolution 201 spectrophotometer (Thermo Fisher Scientific, Hillsboro, OR, USA)). The total content of flavonoids was expressed in milligrams of catechin per gram of sample (mg CT / g sample), after carrying out a suitable calibration curve.

S4. Determination of total antioxidant activity

Briefly, 0.3 mL of hydro alcoholic solution (50/50 v/v) of each extract were added to 1.2 mL of reagent solution (0.6 M H_2SO_4, 28.0 M Na_3PO_4, and 4.0 M $(NH_4)_2MoO_4$). The reaction mixture was incubated at 95°C for 150 min and after cooling to room temperature, the absorbance of the mixture was measured at 695 nm against a control, prepared in the same conditions. By using five different CT standard solutions (8.0, 16.0, 24.0, 32.0, and 40.0 μM), a calibration curve was recorded and the correlation coefficient (R2), slope, and intercept of the regression equation obtained by the method of least-squares were calculated. Each measurement was performed in triplicate and data expressed as means (±SD).

S5. Scavenger activity against DPPH radical

Aqueous solutions of each sample were prepared at different concentrations, and, at 1.0 mL every solution 4.0 mL of purified water and 5.0 mL of an ethanolic solution of DPPH (200 μmol L^{-1}) were added, obtaining a radical concentration of 100 μmol L^{-1}. The mixture was kept at 25°C for 30 minutes and the residual concentration of the DPPH radical DPPH was evaluated with a spectrophotometer at 517 nm. The percentage of inhibition of the DPPH DPPH radical species was calculated according to the following formula:

$$\text{Inhibition (\%)} = (A_0 - A_1)/A_0 \times 100$$

where A_0 is the absorbance of the control prepared under the same conditions using 5 mL of purified water, and A_1 is the absorbance of each examined sample. The scavenging activity on the lipophilic DPPH radical was expressed in terms of IC_{50}.

S6. Determination of scavenging effect on the ABTS radical cation.

ABTS was dissolved in water to reach 7.0 mM concentration. ABTS radical cation ($ABTS^{\cdot+}$) was produced by reacting ABTS stock solution with 2.45 mM potassium persulfate (final concentration) and allowing the mixture to stand in the dark at room temperature for 12-16 h before use. Because ABTS and potassium persulfate react at a ratio of 1:0.5, this leads to an incomplete oxidation of the ABTS. Oxidation of the ABTS started immediately, but the absorbance was not maximal and stable until more than 6 hours. Finally, the concentration of the resulting ABTS.+ solution was adjusted to an absorbance of 0.970±0.020 at 734 nm. The radical was stable in this form for more than two days when stored in the dark at room temperature. In order to evaluate the scavenging effect of the carob leave extracts, enriched kefir and gluten-free bread extracts, 500 μL of an aqueous solution of each sample were mixed with 2.0 mL of the ABTS radical solution. The mixture, was then incubated in a water bath at 37°C and protected from light for 5 min. The decrease of absorbance at 734 nm was measured at the endpoint of 5 min. The scavenging activity was expressed as a percentage of scavenging activity on the ABTS radical according to equation (1). All samples were assayed in triplicate and data expressed as mean (±SD).
